# Supplementary material for: Using ISARIC 4C mortality score to predict dynamic changes in mortality risk in COVID-19 patients during hospital admission
Source: PLoS One. 2022 Oct 12;17(10):e0274158. doi: 10.1371/journal.pone.0274158 (PMC9555674; doi:10.1371/journal.pone.0274158)
Supplement: S2 Fig — (DOCX) [file pone.0274158.s002.docx]

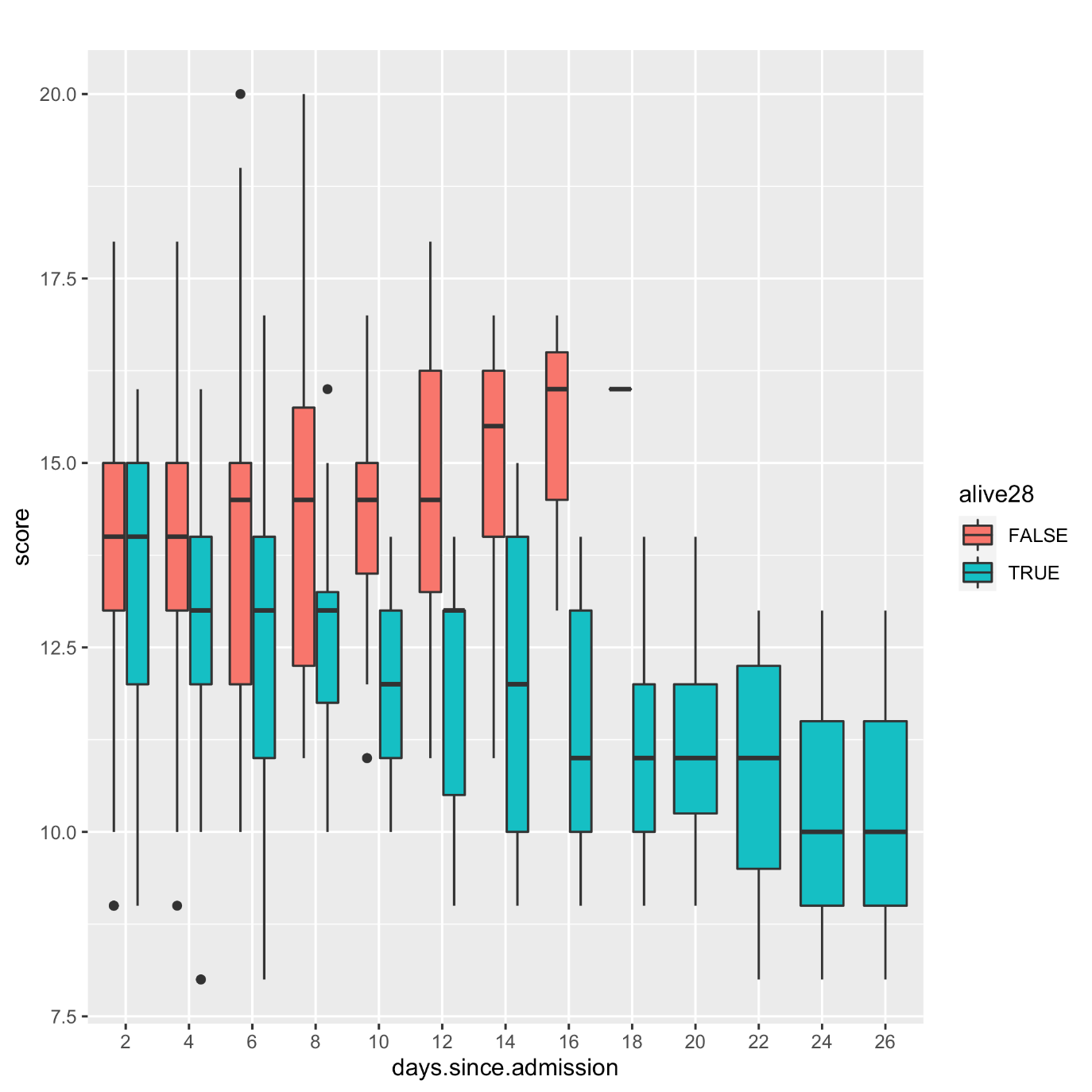
 **Figure S2: showing mean and range of ISARIC 4C scores at 48-hour intervals amongst survivors (turquoise) and decedents (red) with a score of 15 at admission**
