# Supplementary material for: Using ISARIC 4C mortality score to predict dynamic changes in mortality risk in COVID-19 patients during hospital admission
Source: PLoS One. 2022 Oct 12;17(10):e0274158. doi: 10.1371/journal.pone.0274158 (PMC9555674; doi:10.1371/journal.pone.0274158)
Supplement: S3 Fig — (DOCX) [file pone.0274158.s003.docx]

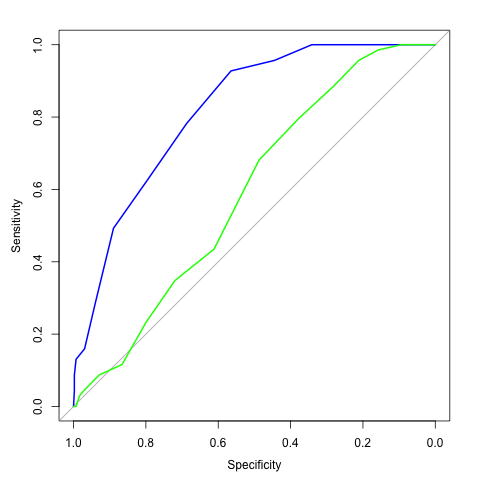


**Figure S3: receiver operating characteristic curves comparing sensitivity and specificity of mortality risk using 4C score at admission (green) and at day 16 (blue)**

s
