# Supplementary material for: Using ISARIC 4C mortality score to predict dynamic changes in mortality risk in COVID-19 patients during hospital admission
Source: PLoS One. 2022 Oct 12;17(10):e0274158. doi: 10.1371/journal.pone.0274158 (PMC9555674; doi:10.1371/journal.pone.0274158)
Supplement: S1 Table — (DOCX) [file pone.0274158.s004.docx]

| **Variable** | **Level** | **Patients with admission 4C = 5**  **(n, %)** | **Patients with admission 4C = 10 (n, %)** | **Patients with admission 4C = 15 (n, %)** |
| --- | --- | --- | --- | --- |
| Sex | Female | 201 (42.3) | 238 (39.8) | 47 (34.6) |
|  | Male | 274 (57.7) | 360 (60.2) | 89 (65.4) |
| Age | Under 50 | 136 (28.6) | 3 (0.5) | 0 (0.0) |
|  | 50-59 | 210 (44.2) | 21 (3.5) | 0 (0.0) |
|  | 60-69 | 129 (27.2) | 147 (24.6) | 9 (6.6) |
|  | 70-79 | 0 (0.0) | 201 (33.6) | 29 (21.3) |
|  | 80 and over | 0 (0.0) | 226 (37.8) | 98 (72.1) |
| Co-morbidities | 0 | 329 (69.3) | 176 (29.4) | 18 (13.2) |
|  | 1 | 72 (15.2) | 114 (19.1) | 17 (12.5) |
|  | 2 or more | 74 (15.6) | 308 (51.5) | 101 (74.3) |
| Respiratory rate (breaths per minute) | 0 to 19 | 146 (32.5) | 189 (34.2) | 36 (26.7) |
|  | 20 to 29 | 223 (49.7) | 306 (55.3) | 78 (57.8) |
|  | 30 or more | 80 (17.8) | 58 (10.5) | 21 (15.6) |
| Oxygen saturation (%) | Less than 92 | 229 (83.0) | 306 (84.5) | 63 (73.3) |
|  | 92 or more | 47 (17.0) | 56 (15.5) | 23 (26.7) |
| Glasgow Coma Scale  (score out of 15) | 15 | 434 (96.9) | 481 (87.0) | 60 (44.8) |
|  | Less than 15 | 14 (3.1) | 72 (13.0) | 74 (55.2) |
| Urea (mmol/L) | Less than 7 | 370 (92.5) | 293 (57.6) | 5 (3.7) |
|  | 7 to 14 | 22 (5.5) | 182 (35.8) | 34 (25.4) |
|  | Greater than 14 | 8 (2.0) | 34 (6.7) | 95 (70.9) |
| C-reactive protein (mmol/L) | less than 50 | 107 (29.7) | 134 (29.4) | 11 (8.5) |
|  | 50-99 | 112 (31.1) | 134 (29.4) | 35 (26.9) |
|  | 100 or greater | 141 (39.2) | 188 (41.2) | 84 (64.6) |

**Table S1: baseline characteristics on score components at admission in patient grouped by admission 4C scores of 5, 10 and 15**
