# Supplementary material for: Using ISARIC 4C mortality score to predict dynamic changes in mortality risk in COVID-19 patients during hospital admission
Source: PLoS One. 2022 Oct 12;17(10):e0274158. doi: 10.1371/journal.pone.0274158 (PMC9555674; doi:10.1371/journal.pone.0274158)
Supplement: S2 Table — (DOCX) [file pone.0274158.s005.docx]

| Variable | Level | Low  (n=84/184) | | Intermediate  (n=235/562) | | High  (n=414/835) | | Very High  (n=56/110) | |
| --- | --- | --- | --- | --- | --- | --- | --- | --- | --- |
|  |  | **Admission** | **Day 8** | **Admission** | **Day 8** | **Admission** | **Day 8** | **Admission** | **Day 8** |
| Sex | Female | 39 (46.4) |  | 109 (46.4) |  | 182 (44.0) |  | 13 (23.2) |  |
|  | Male | 45 (53.6) |  | 126 (53.6) |  | 232 (56.0) |  | 43 (76.8) |  |
| Age | Under 50 | 73 (86.9) |  | 21 (8.9) |  | 0 (0.0) |  | 0 (0.0) |  |
|  | 50-59 | 11 (13.1) |  | 59 (25.1) |  | 5 (1.2) |  | 0 (0.0) |  |
|  | 60-69 | 0 (0.0) |  | 90 (38.3) |  | 44 (10.6) |  | 0 (0.0) |  |
|  | 70-79 | 0 (0.0) |  | 43 (18.3) |  | 121 (29.2) |  | 4 (7.1) |  |
|  | 80 and over | 0 (0.0) |  | 22 (9.4) |  | 244 (58.9) |  | 52 (92.9) |  |
| Number of co-morbidities | 0 | 70 (83.3) |  | 152 (64.7) |  | 94 (22.7) |  | 3 (5.4) |  |
|  | 1 | 7 (8.3) |  | 32 (13.6) |  | 64 (15.5) |  | 6 (10.7) |  |
|  | 2 or more | 7 (8.3) |  | 51 (21.7) |  | 256 (61.8) |  | 47 (83.9) |  |
| Respiratory rate (breaths per minute) | 0 to 19 | 32 (38.1) | 65 (77.4) | 110 (46.8) | 181 (77.0) | 209 (50.5) | 323 (78.0) | 32 (57.1) | 22 (39.3) |
|  | 20 to 29 | 47 (56.0) | 19 (22.6) | 118 (50.2) | 50 (21.3) | 195 (47.1) | 86 (20.8) | 23 (41.1) | 34 (60.7) |
|  | 30 or more | 5 (6.0) | 0 (0.0) | 7 (3.0) | 4 (1.7) | 10 (2.4) | 5 (1.2) | 1 (1.8) | 0 (0.0) |
| Oxygen saturation (%) | Less than 92 | 65 (77.4) | 83 (98.8) | 183 (77.9) | 214 (91.1) | 358 (86.5) | 346 (83.6) | 49 (87.5) | 29 (51.8) |
|  | 92 or more | 19 (22.6) | 1 (1.2) | 52 (22.1) | 21 (8.9) | 56 (13.5) | 68 (16.4) | 7 (12.5) | 27 (48.2) |
| Glasgow Coma Scale  (score out of 15) | 15 | 81 (96.4) | 83 (98.8) | 211 (89.8) | 219 (93.2) | 294 (71.0) | 293 (70.8) | 24 (42.9) | 18 (32.1) |
|  | Less than 15 | 3 (3.6) | 1 (1.2) | 24 (10.2) | 16 (6.8) | 120 (29.0) | 121 (29.2) | 32 (57.1) | 38 (67.9) |
| Urea (mmol/L) | Less than 7 | 77 (91.7) | 57 (67.9) | 157 (66.8) | 139 (59.1) | 160 (38.6) | 134 (32.4) | 7 (12.5) | 3 (5.4) |
|  | 7 to 14 | 7 (8.3) | 27 (32.1) | 53 (22.6) | 87 (37.0) | 162 (39.1) | 213 (51.4) | 21 (37.5) | 19 (33.9) |
|  | Greater than 14 | 0 (0.0) | 0 (0.0) | 25 (10.6) | 9 (3.8) | 92 (22.2) | 67 (16.2) | 28 (50.0) | 34 (60.7) |
| C-reactive protein (mmol/L) | less than 50 | 36 (42.9) | 73 (86.9) | 87 (37.0) | 163 (69.4) | 165 (39.9) | 197 (47.6) | 20 (35.7) | 7 (12.5) |
|  | 50-99 | 16 (19.0) | 8 (9.5) | 66 (28.1) | 44 (18.7) | 120 (29.0) | 131 (31.6) | 17 (30.4) | 14 (25.0) |
|  | 100 or greater | 32 (38.1) | 3 (3.6) | 82 (34.9) | 28 (11.9) | 129 (31.2) | 86 (20.8) | 19 (33.9) | 35 (62.5) |

**Table S2: 4CD components (n, %) in patients with low (0-3), intermediate (4-8), high (9-14) and very high (15+) scores at day 8, for those with complete data at both time points in all measurement**
